# Risk of Serious Infections in Patients Treated With Biologic or Targeted‐synthetic Disease Modifying Antirheumatic Drugs in Qatar

**DOI:** 10.1002/iid3.70195

**Published:** 2025-04-14

**Authors:** Sreethish Sasi, Hamad Abdel Hadi, Masautso Chaponda, Reem El Ajez, Mohamed Ataelmanan, Sief Khasawneh, Hind Saqallah, Maisa Ali, Nabeel Abdulla, Javed Iqbal, Ali S. Omrani, Muna Al Maslamani, Abdullatif Al‐Khal

**Affiliations:** ^1^ Department of Medicine Communicable Diseases Center, Infectious Diseases Division, Hamad Medical Corporation Doha Qatar; ^2^ Department of Medicine Infectious Diseases Division, Al‐Wakra Hospital, Hamad Medical Corporation Doha Qatar; ^3^ Department of Pharmacy Hamad Medical Corporation Doha Qatar; ^4^ Department of Internal Medicine Hamad Medical Corporation Doha Qatar; ^5^ Department of Internal Medicine Rheumatology Division, Hamad Medical Corporation Doha Qatar; ^6^ Department of Nursing Communicable Diseases Center, Hamad Medical Corporation Doha Qatar; ^7^ College of Medicine Qatar University Doha Qatar

**Keywords:** biologic, DMARD, immunocompromised, TNF‐alpha inhibitor, tofacitinib

## Abstract

**Background:**

Biologic and targeted‐synthetic disease‐modifying antirheumatic drugs (b/tsDMARDs), are pivotal in the management of autoimmune‐inflammatory disorders, acting by suppressing pathological immune activation. Because of associated immune dysfunction, opportunistic or serious infections (SIs), and latent disease reactivation is frequently reported. This study aimed to investigate the epidemiology, risk factors, and outcomes of SIs in patients treated with b/tsDMARDs in Qatar.

**Methods:**

A retrospective cohort study was conducted at Hamad Medical Corporation, including all the patients treated with one of 10 b/tsDMARDs, between January 2017 and July 2021. Besides descriptive statistics, the Chi‐square test and Kaplan–Meyer survival analysis were used for statistical analysis.

**Results:**

Out of 1092 patients, 86 (7.9%) had SIs, with an incidence rate of 39.4 per 1000 patient years. Mean duration of onset was 10.8 months post‐initiation of therapy. Younger age groups (18–52 years) were predominantly affected. A significant association was observed between the primary diagnosis (rheumatological followed by gastrointestinal, neurological, and dermatological disorders) and the occurrence of SIs (*χ*² = 9.512, *p* < 0.050). Adalimumab and infliximab had a higher risk of SIs compared to other b/tsDMARDs. There was no significant difference between TNF‐inhibitors and others. Ocrelizumab was significantly associated with incidence of COVID‐19 SIs (*χ*² = 16.84, *p* = 0.0000408), and etanercept with *Staphylococcus aureus* SIs (*χ*² = 17.51, *p* = 0.0000285). Predominant infection sites were skin–soft tissue and respiratory tract. Most of the SIs were secondary to either bacteria (43%) or viruses (17.4%). The mean duration of hospitalization was 9 days, and 7% of patients required critical care, with no recorded 90‐day mortality.

**Conclusions:**

Patients with inflammatory conditions managed with b/tsDMARDs are at significant risk of SIs, which necessitate appropriate patient selection weighing benefits and risks, as well as careful long‐term management that include patient education and relevant preventive therapy.

## Graphical abstract

This study investigates the risk of serious infections (SIs) in patients treated with biologic or targeted‐synthetic disease‐modifying antirheumatic drugs (b/tsDMARDs) in Qatar. Out of 1092 patients, 86 (7.9%) experienced SIs, with adalimumab and infliximab associated with higher SI rates. Our findings highlight the necessity for vigilant monitoring and tailored management strategies to mitigate infection risks in patients on b/tsDMARD therapy.

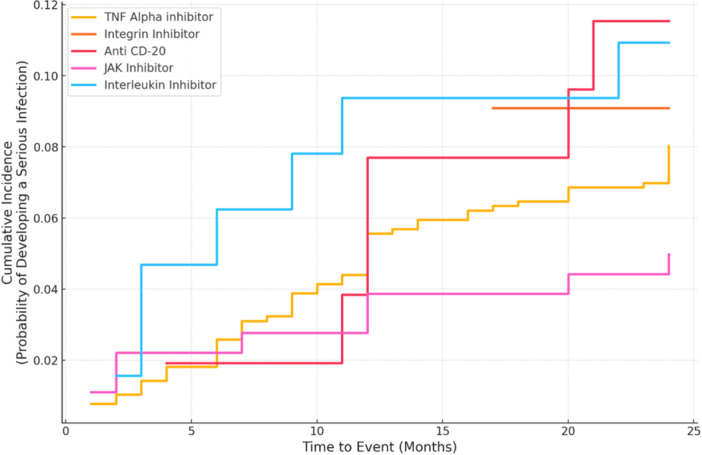

**Abbreviations**
AIIRD: Autoimmune Inflammatory Rheumatic Diseases
AS: Ankylosing Spondylitis
bDMARDs: Biologic Disease‐Modifying Antirheumatic Drugs
CD: Crohn's Diseases
CDC: Centers for Disease Control and Prevention
csDMARDs: Conventional Synthetic Disease‐Modifying Antirheumatic Drugs
DMARDs: Disease‐Modifying Antirheumatic Drugs
EPR: Electronic Patient Records
EULAR: European League Against Rheumatism
HPV: Human Papillomavirus
JAK: Janus Kinase
MS: Multiple Sclerosis
ORs: Odds Ratios
PCV13: Pneumococcal Conjugate Vaccine 13
PPSV23: Pneumococcal Polysaccharide Vaccine 23
PsA: Psoriatic Arthritis
RA: Rheumatoid Arthritis
SIs: Serious Infections
SLE: Systemic Lupus Erythematosus
SPSS: Statistical Package for the Social Sciences
TNF: Tumor Necrosis Factor
tsDMARDs: Targeted Synthetic Disease‐Modifying Antirheumatic Drugs
UC: Ulcerative Colitis

## 1 Introduction

For many decades, the underlying causes of debilitating rheumatological conditions like rheumatoid arthritis (RA), systemic lupus erythematosus (SLE), ankylosing spondylitis (AS), gastrointestinal disorders such as ulcerative colitis (UC), and Crohn's disease (CD), along with various inflammatory dermatological and neurological conditions, have remained elusive mysteries. Nevertheless, the common thread linking these systemic diseases lies in the abnormal activation of immune responses, leading to damage in end organs or tissues [1]. Central to addressing this immune dysregulation is the need for immune suppression and modulation to mitigate or prevent subsequent complications [2]. Of these directed therapies, disease‐modifying antirheumatic drugs (DMARDs) are pivotal in the management of auto‐immune inflammatory conditions, fundamentally targeting the immune system, altering the disease course to prevent further damage [3]. DMARDs are broadly categorized into three classes: conventional synthetic (csDMARDs), targeted synthetic (tsDMARDs), and biologic (bDMARDs) [4]. csDMARDs, including methotrexate, sulfasalazine, and hydroxychloroquine, are the cornerstone of RA treatment due to their established efficacy and safety profiles [3]. tsDMARDs and bDMARDs represent newer classes designed to target specific pathways in the inflammatory process [3, 4, 5]. tsDMARDs, such as Janus kinase (JAK) inhibitors (e.g., tofacitinib and baricitinib), block intracellular signaling pathways, while bDMARDs, including tumor necrosis factor (TNF)‐alpha inhibitors (e.g., infliximab, etanercept, adalimumab, golimumab, and certolizumab) and non‐TNF inhibitors (e.g., tocilizumab, natalizumab, ocrelizumab, secukinumab, rituximab, and abatacept), target extracellular molecules or cell surface receptors [4]. TNF‐alpha inhibitors (TNFi) reduce inflammation and halt disease progression by blocking the action of TNF‐alpha, a key cytokine in the inflammatory process, whereas non‐TNF biologics (non‐TNFi) disrupt other components of the immune response, offering alternatives for patients who do not respond to or tolerate TNF inhibitors [4, 6]. bDMARDs have expanded the therapeutic landscape for various chronic conditions beyond rheumatology [7], addressing the unmet needs in dermatology [8, 9], gastroenterology [10], and neurology [11]. In rheumatology, biologics have dramatically improved the prognosis of RA, AS, and psoriatic arthritis (PsA) by inhibiting pro‐inflammatory cytokines or cellular interactions necessary for autoimmunity [3, 7, 12, 13, 14, 15]. Their use is also crucial in conditions like SLE, Sjogren's syndrome, and other connective tissue diseases [16]. Furthermore, in pediatric populations, biologics have altered the course of chronic juvenile arthritis, providing symptom relief, and preventing joint damage [10, 17]. In dermatology, biologics have been transformative for conditions such as psoriasis, yielding significant skin clearance and improving quality of life [8]. They also play a role in the management of hidradenitis suppurativa or acne inversa and are being explored for vitiligo, alopecia areata, and scleroderma [10, 18]. In gastroenterology, biologics targeting TNF‐alpha, integrins, or interleukins have become mainstays for treating CD and UC [19]. In inflammatory neurological conditions, biologics reduce the frequency of relapses in multiple sclerosis (MS) [11].

The study of the risk of serious infections (SIs) associated with the use of bDMARDs or tsDMARDs is of paramount importance. While b/tsDMARDs are revolutionizing the treatment paradigms with substantial benefits, they come with a caveat of possible adverse effects, including the increased risk of infections due to their immunomodulatory actions. Observational data, such as the NOR‐DMARD study [14], highlights an increased risk of SIs with TNFi therapy in RA compared to csDMARDs, indicating a need for careful patient monitoring. This study focused on RA and PsA patients starting TNFi treatment and reported the crude incidence rates (IRs) for SIs as 4.17 (95% CI: 3.52–4.95) in patients with RA and 2.16 (95% CI: 1.66–2.81) in patients with PsA. On the other hand, patients with PsA treated with TNFi exhibit a lower risk of SIs when compared to those with RA, suggesting disease‐specific factors also contribute to the infection risk profile [14]. In Saudi Arabia, a study conducted at King Abdul‐Aziz University Hospital showed that patients with RA using csDMARDs, either alone or in combination with biologics, had a substantial risk of developing urinary tract infections (UTIs) [20]. Such data underscore the need for diligent infection surveillance protocols during b/tsDMARD therapy. This is particularly important in regions where infections such as tuberculosis have a high prevalence, which could be exacerbated by the immunosuppressive nature of these therapies [6, 14, 17, 21, 22, 23, 24]. These differences in infection risk between diseases stress the importance of personalized treatment approaches and highlight the utility of large‐scale registries and databases in refining our understanding of b/tsDMARD safety profiles. This knowledge is not only vital for clinicians when weighing the benefits against potential risks for individual patients but also for informing guidelines and patient education on the importance of infection monitoring and prevention strategies during treatment with b/tsDMARDs.

This study aims to fill a critical gap in our current understanding of infection risks associated with DMARD treatments in the Middle Eastern region. The objective is to elucidate the epidemiology and risk factors for SIs in patients undergoing b/tsDMARD therapy. The study examined the incidence of SIs with an emphasis on comparing TNFi vs. non‐TNFi biologicals. What this study adds to the literature is a comprehensive, real‐world analysis from Qatar that can be leveraged to inform global research, advancing our understanding of DMARD‐related infection risks.

## 2 Materials and Methods

Hamad Medical Corporation (HMC) is the primary healthcare provider for secondary and tertiary care for almost three million people in the State of Qatar [25]. It acts through 14 general and specialized care hospitals with bed capacity of almost 2500 and multiple specialized outpatient services and national electronic patient records (EPR) that allows for accurate identification [25]. This study was conducted in accordance with the ethical principles outlined in the Declaration of Helsinki and was approved by the Medical Research Center at HMC (approval number: MRC‐01‐22‐691). This study was exempted from obtaining informed consent by the Institutional Review Board (IRB), as it involved only a retrospective review of de‐identified data from EPR. This retrospective observational study included all patients above 14 years of age, from outpatient rheumatology, gastroenterology, dermatology, and neurology services, who commenced bDMARDs and tsDMARD treatments between January 1, 2017 and July 31, 2021. The inclusion of patients aged 14–17 years was based on their management under adult treatment protocols at our institution. In Qatar healthcare system, all the patients aged 14 years and above are treated by adult medicine or its sub‐specialties, and the IRB permits their inclusion in studies exempted from the requirement of obtaining informed consent. Patients started on a predefined set of bDMARDs, which included non‐TNFi bDMARDs (ocrelizumab, tocilizumab, secukinumab, natalizumab), TNFi bDMARDs (etanercept, adalimumab, infliximab, certolizumab, golimumab), and the tsDMARD tofacitinib during this time period, were identified from pharmacy EPR. Eligible study participants should have been treated with the respective b/tsDMARD for 6 months and have had a follow‐up record of at least 2 years in their EPR. Exclusion criteria comprised patients who had been treated with glucocorticoids for more than 3 months or csDMARDs for more than a year, alongside b/ts‐DMARDs. Patients who received any dose of glucocorticoids as maintenance therapy, along with the DMARDs, for the disease condition under consideration, for at least 3 months, irrespective of whether administered orally or intravenously, were excluded from the study. Those on multiple b/tsDMARDs during the follow‐up, lacking EPR documentation or sufficient follow‐up data, diagnosed with malignancies, residing in nursing homes or hospitalized long‐term were also excluded. Rituximab was excluded as it was primarily used in cancer patients, while abatacept, baricitinib, and upadacitinib were excluded due to insufficient patient numbers meeting the study's inclusion and exclusion criteria. During the study period, biosimilars were not widely available or utilized in Qatar, and none of the drugs included in this study were biosimilars. In Qatar, patients with inflammatory conditions are treated by specialists based on clinical criteria and the treating physician's discretion. While there are no specific national guidelines for initiating DMARDs, treatment decisions follow international standards, such as European League Against Rheumatism (EULAR) recommendations for RA [3] and ECCO guidelines for UC [26].

SIs were defined as those episodes of infections associated with hospitalization for more than 48 h, parenteral antibiotic therapy, or death [27]. All the patients diagnosed to have active tuberculosis at any site were also considered to have SIs even if not associated with the above criteria. This study utilized data extracted retrospectively from Qatar's unified EPR system, established in 2015, which documents all patient encounters. The review covered patients diagnosed with inflammatory conditions, from their initial specialty clinic visit through the initiation of biologics and up to 2 years posttreatment. Key information, including laboratory results, comorbidities, infection spectrum, treatment outcomes, smoking status, diagnosis, and illness duration, was comprehensively analyzed from the EMRs.

### 2.1 Statistical Analysis

All the statistical analyses were performed using IBM Corp. Released 2019. IBM SPSS Statistics for Windows, Version 26.0 (Armonk, NY: IBM Corp.). Descriptive statistics, including means and standard deviations for continuous variables and frequencies with percentages for categorical variables, were utilized to summarize demographic and clinical characteristics of the study population. The incidence rates (IR) of SIs were calculated per 1000 patient‐years. Chi‐square tests were employed to identify associations between categorical variables such as age, gender, smoking status, primary diagnosis, and specific biologic or targeted‐synthetic DMARDs. A *p*‐value of less than 0.05 was considered statistically significant. The Kaplan–Meier method was used to estimate survival curves for the time to first SI event, and differences between groups were assessed using the log‐rank test. To address missing data, multiple imputation techniques were applied, which enhanced statistical power and provided a more robust analysis. Logistic regression, with regularization to handle multicollinearity, was used to identify predictors of SIs. Covariates included in the model were patient demographics, comorbidities (e.g., Charlson Comorbidity Index), DMARD type (biologic vs. targeted synthetic), and mechanism of action (e.g., TNF inhibitors, JAK inhibitors). Odds ratios (ORs) were calculated to assess the relative risk of SIs associated with these variables.

## 3 Results

Strict exclusion criteria were enforced to minimize the influence of other medications on the incidence of SIs attributed to DMARDs. The initial cohort included 6800 patients, of whom 1400 had RA. After applying stringent exclusion criteria, the final cohort was reduced to 1092 patients. A subset of 665 patients included individuals with a variety of rheumatological conditions, such as RA, AS, PsA, mixed CTDs, peripheral SpA, seronegative arthritis, adult‐onset Still's disease, SLE, Sjögren's syndrome, granulomatous uveitis, inflammatory arthropathy, Takayasu arteritis, and chronic juvenile arthritis. A significant number of RA patients were excluded because they were simultaneously receiving both synthetic and b‐DMARDs. This rigorous approach ensured that the analysis accurately reflected the risk of SIs specifically associated with b/tsDMARDs. Dermatological conditions included in the study cohort were psoriasis, hidradenitis/acne, vitiligo, alopecia areata, alopecia universalis, erythema nodosum, and scleroderma/morphea. The gastrointestinal conditions were CD and UC, while the neurological condition was MS. Among the 1092 patients treated with b/tsDMARDs, 86 (7.9%) experienced SIs. The demographic characteristics of these patients are outlined in Table 1. The SI incidence rate (IR) was 39.4 per 1000 patient‐years. The mean age of patients who developed SIs was 42 years, indicating that these events were not limited to older populations.

**Table 1:** Patient demographics, primary diagnoses, and b/tsDMARD characteristics associated with serious infections.

Variable	Characteristics	Serious infection		Total (1092)	*χ*² *p*
		No (1006, 92.1%)	Yes (86, 7.9%)		
**Age (years)**	Below 18	6 (0.5%)	1 (0.1%)	7 (0.6%)	16.336 0.003***
	18–35	324 (29.7%)	30 (2.7%)	354 (32.4%)	
	36–52	468 (42.9%)	37 (3.4%)	505 (46.2%)	
	53–70	190 (17.4%)	11 (1.0%)	201 (18.4%)	
	Above 70	18 (1.6%)	7 (0.6%)	25 (2.3%)	
**Gender**	Female	509 (46.6%)	49 (4.5%)	558 (51.1%)	1.291 0.256
	Male	497 (45.5%)	37 (3.4%)	534 (48.9%)	
**Smoking**	Yes	107 (9.8%)	14 (1.3%)	121 (11.1%)	2.560 0.11
	No	899 (82.3%)	72 (6.6%)	971 (88.9%)	
**Primary diagnosis**	Dermatological	179 (16.4%)	7 (0.6%)	186 (17.0%)	9.512 0.050***
	Gastrointestinal	141(12.9%)	19 (1.7%)	160(14.7%)	
	Neurological	66 (6.0%)	8 (0.7%)	74(6.8%)	
	Rheumatological	611 (56.0%)	52 (4.8%)	663(60.7%)	
	Others	9 (0.8%)	0 (0.0%)	9 (0.8%)	
**Duration of illness** before starting the b/tsDMARD (years)	<3	406 (37.2%)	32 (2.9%	438 (40.1%)	1.72 0.633
	3–5	209 (19.1%)	23 (2.1%)	232 (21.2%)	
	5–10	195(17.9%)	16 (1.5%)	211 (19.3%)	
	>10	196 (17.9%)	15 (1.4%)	211 (19.3%)	
**Name of the b/ts‐DMARD**	Adalimumab	303 (27.7%)	17 (18.4%)	320 (29.3%)	18.982 < 0.025*
	Certolizumab	78 (7.1%)	10 (10.9%)	88 (8.1%)	
	Etanercept	100 (9.2%)	9 (9.8%)	109 (10.0%	
	Golimumab	148 (13.6%)	10 (10.9%)	158 (14.5%)	
	Infliximab	82 (7.5%)	16 (17.3%)	98 (9.0%)	
	Natalizumab	20 (1.8%)	2 (2.2%)	22 (2.0%)	
	Ocrelizumab	46 (4.2%)	6 (6.5%)	52 (4.8%)	
	Secukinumab	39 (3.9%)	4 (4.3%)	43 (3.9%)	
	Tocilizumab	18 (1.6%)	3 (3.3%)	21 (1.9%)	
	Tofacitinib	172 (15.8%)	9 (9.8%)	181 (16.6%)	
**Class of the DMARD**	Biologic (b)‐DMARD	834 (83%)	77 (83.7%)	911(83.4%)	2.52 0.112
	Semisynthetic (cs)‐DMARD	172 (17%)	9 (16.3%)	181 (16.6%)	
**Type of DMARD**	non–TNF alpha inhibitors	295 (29%)	24 (26%)	319 (29.2%)	0.77 0.781
	TNF alpha inhibitors	711 (71%)	62 (74%)	773 (70.8%)	
**Mechanism of action of the b/ts‐DMARD**	Anti CD‐20	46 (4.6%)	6 (6.5%)	52 (4.8%)	3.959 0.412
	Integrin inhibitors	20 (2%)	2 (2.2%)	22 (2.0%)	
	Interleukin inhibitors	57 (5.7%)	7 (7.6%)	64 (5.9%)	
	JAK inhibitors	172 (17%)	9 (10.4%)	181 (16.6%)	
	TNF‐alpha inhibitors	711 (71%)	62 (72%)	773 (70.8%)	
**Whether csDMARDs were used along with b/ts DMARDS**	No	588 (58.4%)	53 (61.6%)	641(58.7%)	0.33 0.566
	Yes	418 (41.6%)	33(38.4%)	451 (41.3%)	

* *p* < 0.05

*** *p* ≤ 0.005.

### 3.1 Sociodemographic Associations

Age proved to be a significant factor related to SI incidence among those treated with b/tsDMARDs, particularly notable in the 18–52 age group (*χ*² = 16.336, *p* < 0.003). Interestingly, the extremes of age groups, under 18 and above 70, showed lower rates, potentially influenced by their smaller sample sizes. Gender and smoking did not show a significant association with SIs (*p* > 0.05), pinpointing age as a critical risk factor needing close monitoring (Table 1).

### 3.2 Primary Diagnosis and Duration of Illness

A significant association was observed between the primary diagnosis and the occurrence of serious infections (*χ*² = 9.512, *p* < 0.050). Patients with rheumatological conditions had the highest SI rates, followed by those with gastrointestinal, neurological, and dermatological disorders. The duration of illness before starting DMARD therapy did not significantly influence SI rates (*χ*² = 1.72, *p* = 0.632) (Table 1).

### 3.3 Drug Characteristics

**Figure 1:**
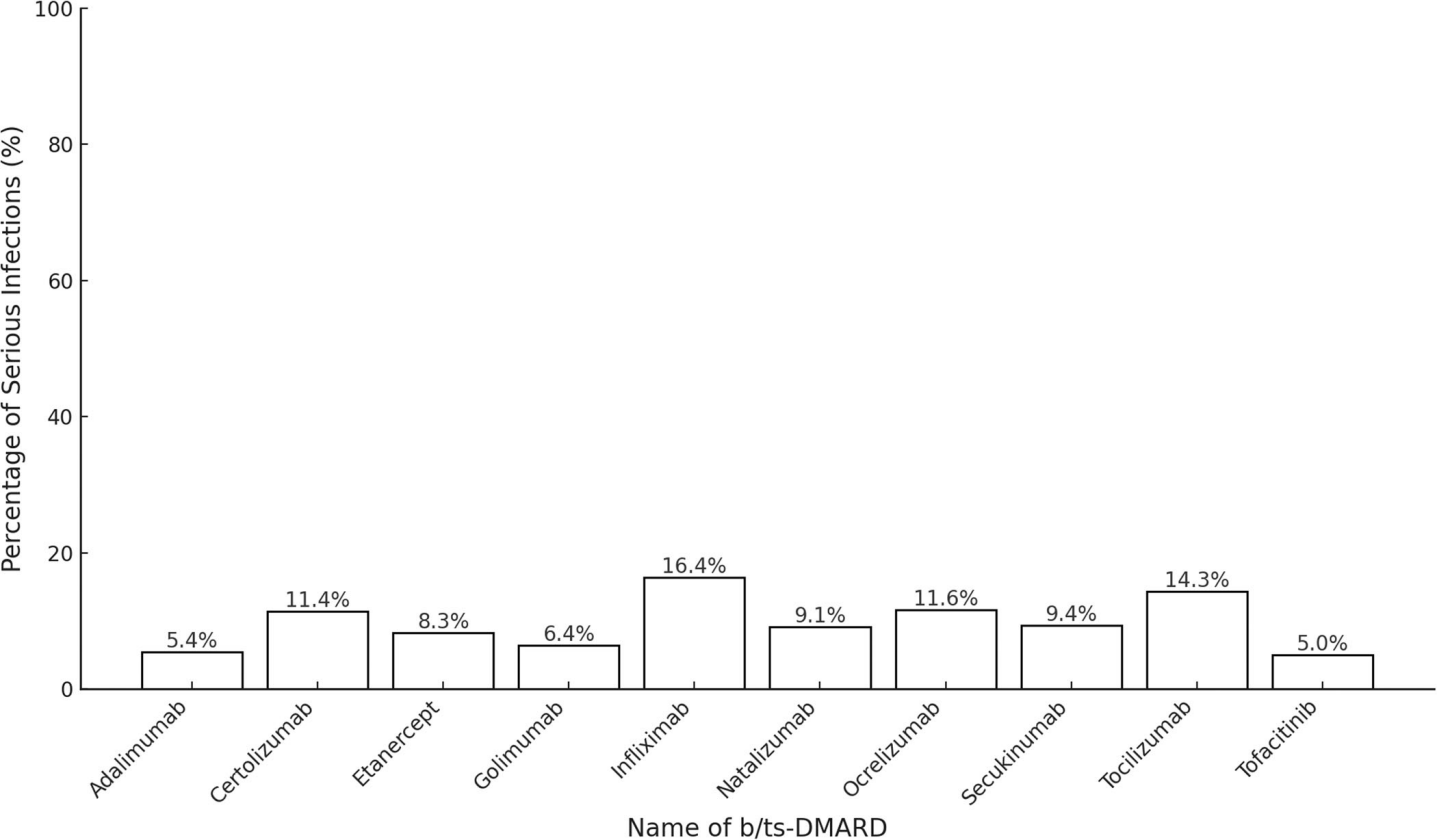
Percentage of serious infections caused by individual b/tsDMARDs.

Neither the class of DMARD (biologic vs. targeted‐synthetic) nor the specific mechanism of action (anti CD‐20, integrin inhibitors, interleukin inhibitors, JAK inhibitors, and TNF‐alpha inhibitors) significantly impacted SI rates. There was no difference between TNFi and non‐TNFi. However, among individual drugs, adalimumab and infliximab were associated with a higher proportion of SIs (*χ*² = 18.982, *p* < 0.025). Percentage of infections caused by individual DMARDs is shown in Figure 1. The concurrent use of csDMARDs with b/tsDMARDs for upto a year did not present a significant risk (*χ*² = 0.33, *p* = 0.566) (Table 1).

### 3.4 The Charlson's Comorbidity Index (CCI)

CCI did not show any significant association with SIs (*χ*² = 3.688, *p* = 0.297), suggesting that comorbidities did not influence the risk of infection in this population.

### 3.5 Hepatitis B & C Serology

Subjects with a negative hepatitis C antibody demonstrated a higher prevalence of SIs compared to those with positive hepatitis C antibody (*χ*² = 9.631, *p* = 0.008). On the contrary, subjects with a positive hepatitis B surface antibody (past infection or immunization) had a higher risk of SIs compared to those with negative HBsAb (*χ*² = 28.95, *p* < 0.000).

### 3.6 Rates of Immunization Before Administration of Biologic

**Figure 2:**
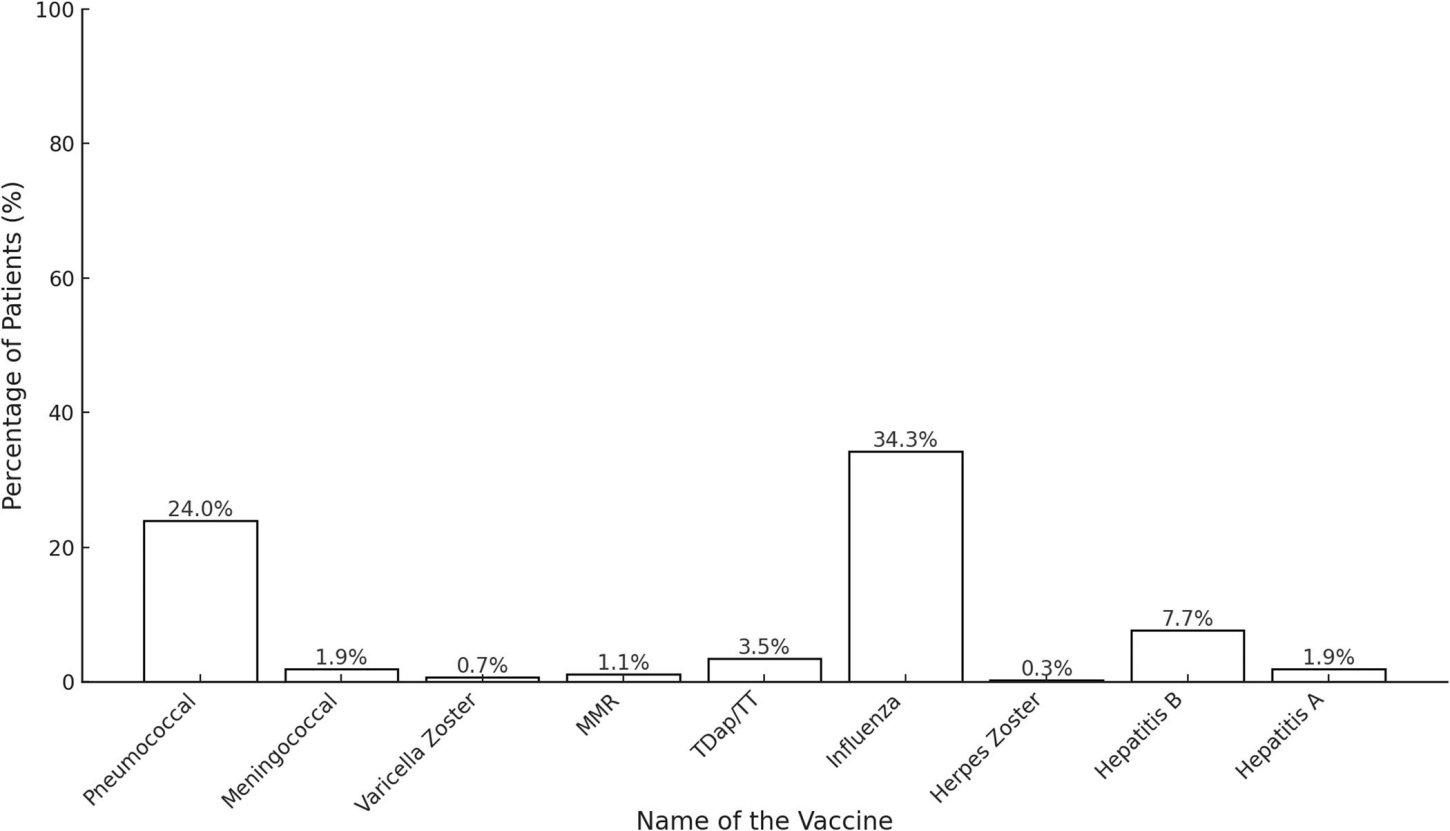
Vaccination coverage before b/tsDMARD therapy (*n* = 1092).

There were low rates prophylactic vaccination among candidates of b/tsDMARD therapy as shown in Figure 2.

### 3.7 Spectrum of Infections

**Figure 3:**
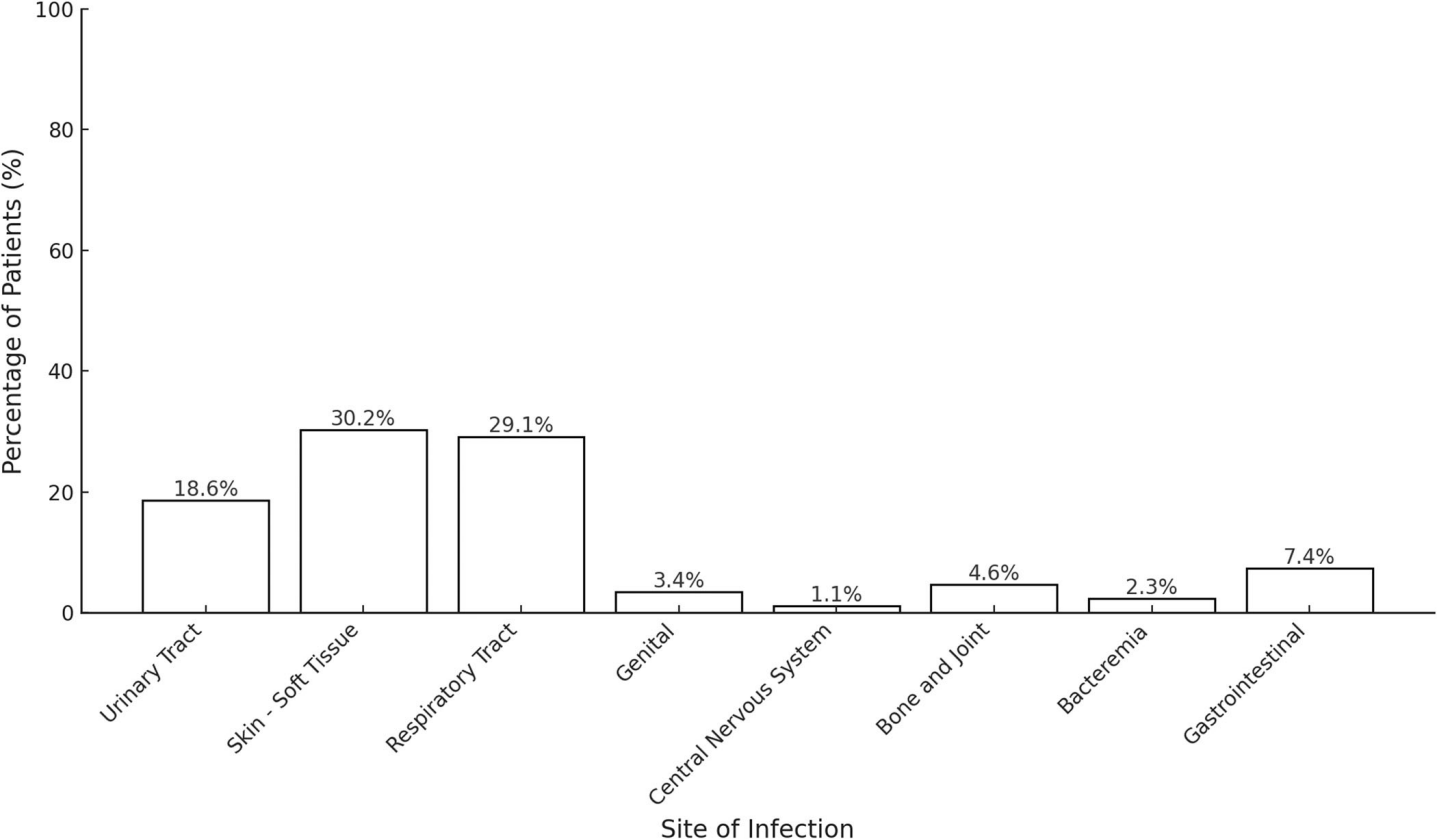
Sites of infection among patients with serious infections (*n* = 86).

The spectrum of infections among the patients was diverse, with skin–soft tissue infections (30.2%) and respiratory tract infections (29.1%) being most common (Figure 3). Bacterial infections (43%) were predominant, particularly *Staphylococcus aureus* (17.4%), while COVID‐19 also represented a notable proportion of viral infections. Methicillin‐resistant *Staphylococcus aureus* (MRSA) represented a third of all *S. aureus* cases, with 5 in 15 samples testing positive. The majority of the gram‐negative *Enterobacterales* (*Escherichia coli* and *Klebsiella pneumoniae*) were resistant, with 6 out of 7 showing the presence of either AmpC or extended‐spectrum beta‐lactamase (ESBL). Cases of active tuberculosis were rare, making up only 3.5% of instances. The vast majority of patients did not need ICU care (91.9%), and 95.3% did not require vasopressor support (Table 2). Adalimumab was not associated with any case of UTI. Ocrelizumab was highly associated with incidence of COVID‐19 SIs (χ² = 16.84, *p* = 0.0000408). Etanercept was highly associated with *S. aureus* SIs (*χ*² = 17.51, *p* = 0.0000285) (Table 3). Subjects who had latent tuberculosis at baseline did not have significantly higher risk of getting active tuberculosis during the b/tsDMARD therapy probably because most of them (96%) received chemoprophylaxis against latent tuberculosis as per WHO guidelines (OR = 6.82, *p* = 0.194).

**Table 2:** Characteristics and microbial spectrum of first episode of serious infection (*n* = 86).

Variables	Characteristics	Frequency (*n* = 86)	Percent
**Site of infection**	Skin–soft tissue	26	30.2
	Respiratory tract	25	29.1
	Gastrointestinal tract	15	17.4
	Urinary tract	10	11.6
	Bone and joint	4	4.7
	Genital tract	3	3.5
	Blood stream	2	2.3
	Central nervous system	1	1.2
**Organism type**	Bacteria	37	43.0
	Virus	15	17.4
	Mycobacterium	3	3.5
	Fungus	2	2.3
	Protozoa	1	1.2
	Pathogen not identified	28	32.6
**Organism name**	Anaerobic bacteria	2	2.3
	Blastocystis	1	1.2
	Candida	2	2.3
	cytomegalovirus	1	1.2
	COVID‐19	12	14.0
	*Escherichia coli*	4	4.7
	*Gonococcus*	1	1.2
	*Hemophilus influenza*	1	1.2
	*Herpes simplex virus*	1	1.2
	*Influenza A*	1	1.2
	*Klebsiella pneumoniae*	3	3.5
	*Mycobacterium tuberculosis complex*	3	3.5
	*Pseudomonas aeruginosa*	2	2.3
	*Salmonella*	1	1.2
	*Serratia marcescens*	1	1.2
	*Staphylococcus aureus*	15	17.4
	*Streptococcus agalactiae*	3	3.5
	*Streptococcus pyogenes*	4	4.7
	Pathogen not identified	28	32.6
**Resistance pattern**	Amp‐C	3	3.5
	ESBL	3	3.5
	MRSA	5	5.8
	No	47	54.7
	Not applicable	28	32.6
**Active TB**	No	83	96.5
	Yes	3	3.5
**Active TB site**	Cervical lymph node	1	1.2
	Pleural	1	1.2
	Pulmonary	1	1.2
	Not applicable	83	95.5
**ICU stay**	No	79	91.9
	Yes	7	8.1
**Vasopressor requirement**	No	82	95.3
	Yes	4	4.7

**Table 3:** b/tsDMARD specific patterns of infection types and microbial pathogens.

		Adalimumab	Infliximab	Certolizumab	Golimumab	Tofacitinib	Etanercept	Ocrelizumab	Secukinumab	Tocilizumab	Natalizumab
	**Total number of subjects**	303	82	78	148	172	100	46	39	18	20
**Organism type**	Bacteria	4	8	5	4	4	7	1	1	1	0
	Not identified	9	6	4	5	0	2	1	2	1	0
	Virus	2	1	0	1	3	0	4	1	1	2
	Mycobacteria	2	1	0	0	0	0	0	0	0	0
	Fungus	0	0	0	0	2	0	0	0	0	0
	Protozoa	0	0	1	0	0	0	0	0	0	0
**Organism name**	*Staphylococcus aureus*	0	0	2	3	0	7	1	1	1	0
	COVID‐19	1	1	0	1	2	0	4	0	1	2
**Site of infection**	Skin–soft tissue	4	3	3	5	4	5	1	1	0	0
	Respiratory tract	6	2	3	1	2	2	4	1	2	2
	Gastrointestinal tract	5	5	2	1	0	0	0	2	0	0
	Urinary tract		4	2	2	1		1	0	0	0
	Bone and joint	1	1	0	0	0	2	0	0	0	0
	Genital tract	1	0	0	0	2	0	0	0	0	0
	Blood stream	0	1	0	0	0	0	0	0	1	0
	Central nervous system	0	0	0	1	0	0	0	0	0	0

### 3.8 Treatment Outcomes

The outcomes were generally favorable, with a 100% hospital discharge rate, zero 30‐day, and low 1‐year mortality (2.3%). Regarding the management of DMARD therapy postinfection, most patients experienced a brief interruption before resuming therapy (38.4%), while others were switched to different classes or continued with the same treatment (Table 4). The majority of patients (92%) did not require intensive care, indicating that most SIs could be managed without the need for critical care interventions.

**Table 4:** Patient outcomes following serious infections and postinfection DMARD management.

Variables	Characteristics	Frequency	Percent
**Outcome of ICU stay (*n*** = **7)**	Step down to medical floor	7	100.0
**Outcome of hospital stay (*n*** = **86)**	Discharge	86	100.0
**30‐day mortality (*n*** = **86)**	No	86	100.0
**1‐year mortality (*n*** = **86)**	No	84	97.7
	Yes	2	2.3
**What happened to the b/tsDMARD during and after the serious infection (*n*** = **86)**	Briefly interrupted and resumed	33	38.4
	Changed to alternative agent of different class	23	26.7
	Changed to alternative agent of same class	7	8.1
	Continued same	19	22.1
	Discontinued	4	4.7

### 3.9 Timing of SIs Posttreatment Initiation

**Figure 4:**
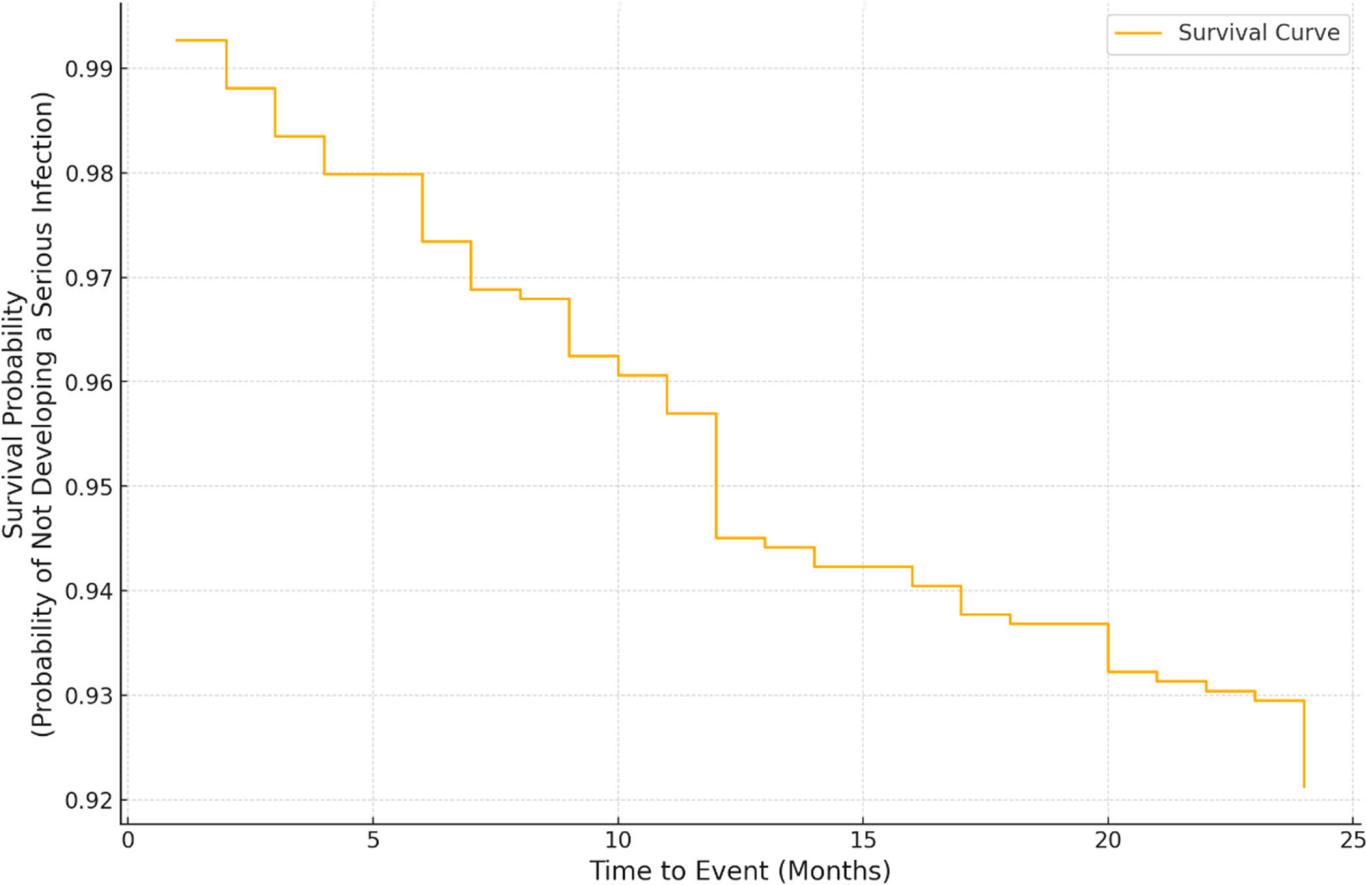
Kaplan–Meier survival curve for the probability of not developing serious infections.

**Figure 5:**
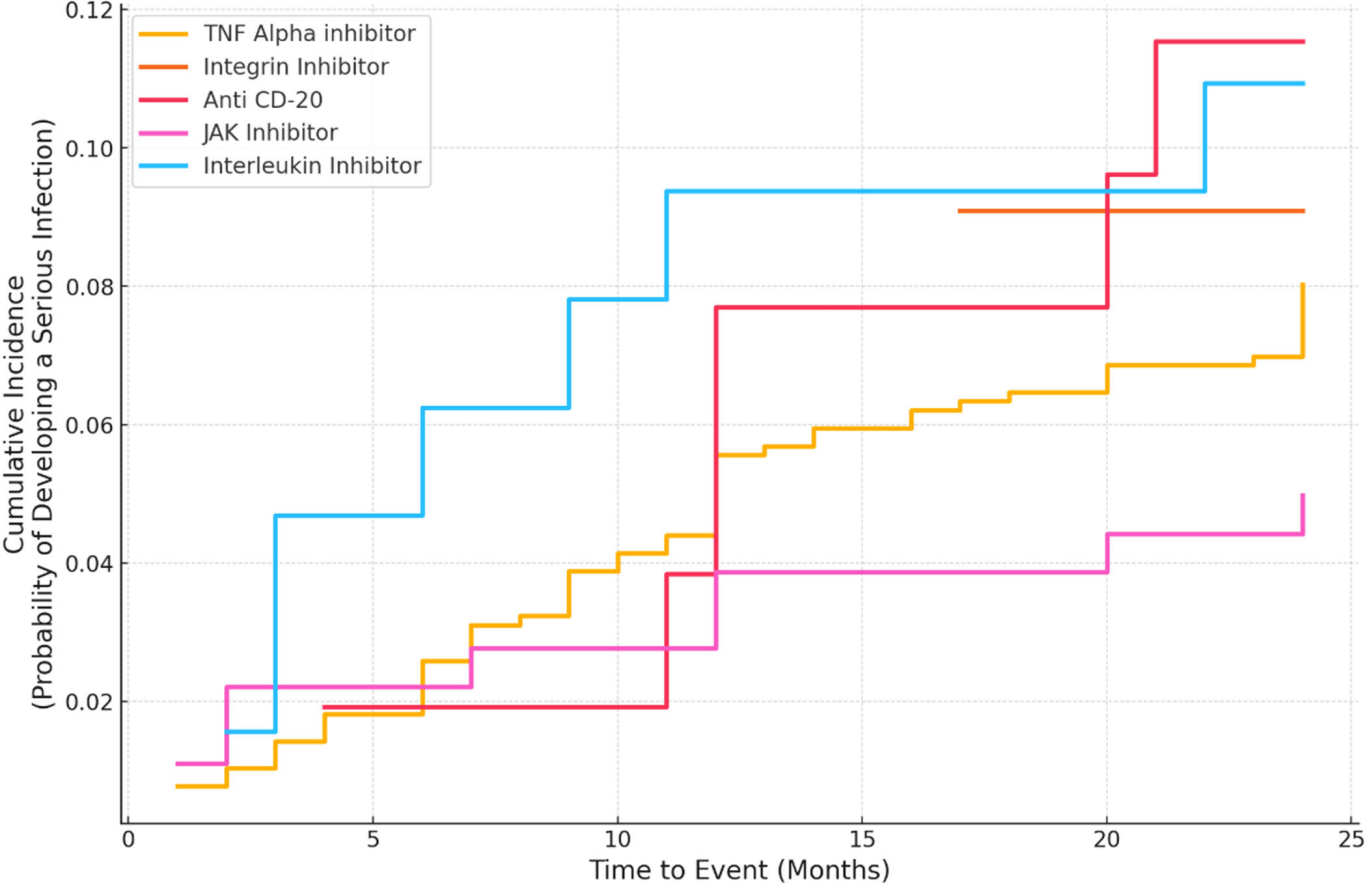
Cumulative incidence curves by mechanism of action: probability of developing serious infections.

SIs typically occurred 10.84 ± 7.3 (range: 1–24) months after initiation of treatment with b/tsDMARDs, highlighting that risks persist well beyond the first 6 months of treatment. A Kaplan–Meier survival analysis graph depicting the probability that a patient has not experienced SI at a specific time point is shown in Figure 4. Regarding hospitalization, the average duration was 7.56 ± 7.93 (range: 2–46) days. A cumulative incidence chart comparing the likelihood of SIs over time for patients receiving different treatments based on their mechanism of action is shown in Figure 5. Interleukin inhibitors show the highest cumulative incidence of SIs over time, suggesting a potentially increased infection risk with their use. Conversely, therapies such as integrin inhibitors and TNF‐alpha inhibitors exhibit lower cumulative incidences, implying a comparatively lower risk. Anti‐CD20 agents and JAK inhibitors demonstrate intermediate risk profiles.

### 3.10 Logistic Regression Model with Regularization

Table 5 presents the results from a logistic regression model with regularization, assessing factors associated with the likelihood of developing SIs. Among comorbidities, obesity and dyslipidemia show significant positive coefficients, indicating a higher risk of SI. Similarly, gastrointestinal and rheumatological conditions among primary disease categories are strongly associated with an increased risk, as reflected by their positive coefficients. Regarding mechanisms of action, TNF‐alpha inhibitors and interleukin inhibitors demonstrate positive coefficients, suggesting an elevated risk of SI. The model indicates that these variables contribute significantly to the overall risk profile for developing SI.

**Table 5:** Predictors of serious infections: Logistic regression analysis with regularization.

Variable		Coefficient	Standard error	Wald statistic	*p* value
Time to event (months)		−0.41	0.05	−161.98	0.04
Comorbidities	Coronary artery disease	−0.328	0.053	−6.140	0.91
	Diabetes mellitus	−0.007	0.039	−0.191	1.618
	Obesity	0.200	0.048	4.195	0.03
	Dyslipidemia	0.342	0.048	7.188	0.001
	Chronic liver disease	0.082	0.045	1.810	2.34
	Hypertension	−0.133	0.057	−2.319	0.9
	Chronic kidney disease	0.021	0.045	0.466	1.068
Primary disease condition	Gastrointestinal conditions	0.366	0.045	8.090	0.02
	Neurological conditions	0.276	1.25	2.516	0.32
	Rheumatological conditions	0.414	0.048	8.668	0.001
	Dermatological conditions	−0.336	0.038	−8.839	0.782
Mechanism of action	JAK inhibitor	−0.239	2.9	−2.3	0.89
	Integrin inhibitor	−0.673	0.043	−15.517	0.78
	TNF‐alpha inhibitor	0.35	0.07	4.87	0.03
	Interleukin inhibitor	0.074	1.89	0.001	0.01
Simultaneous use of synthetic DMARDs		−0.174	0.037	−4.737	0.9

### 3.11 Post Hoc Analysis

Post hoc analyses were conducted for the age categories, primary diagnosis, and name of the b/tsDMARD. The “Above 70” age group showed a significantly higher SI risk compared to “Below 18” and “18–35” groups (*p* < 0.05). Among primary diagnoses, rheumatological conditions contributed most significantly to the SI association. Among individual b/tsDMARDs, adalimumab and infliximab had higher SI rates compared to other drugs (*p* < 0.05).

## 4 Discussion

Biological agents, specifically targeted immunomodulatory drugs, have revolutionized the field of clinical medicine, particularly in the treatment of various autoimmune diseases. They have shown efficacy in managing diseases such as RA, psoriasis, IBD, and MS. However, the increased risk of infections associated with these therapies necessitates a proactive and comprehensive approach to prophylaxis and patient monitoring to ensure treatment safety and efficacy. Multiple studies have already established the increased propensity of b/tsDMARDs to cause SIs in the host [12, 13, 14, 15, 17, 20, 21, 22, 23, 24, 28, 29, 30, 31]. Globally, a systematic review and meta‐analysis by Singh et al. in 2015 [23] highlighted the concern of SIs in patients with RA treated with bDMARDs, indicating an increased risk compared with csDMARDs. However, specific IRs were not provided. 4.2% of the study subjects were hospitalized due to infection in an Italian study by Quartuccio et al. in 2018 [17], among patients suffering from chronic inflammatory autoimmune diseases treated with biologics. Aldauig et al. described the spectrum of infections in rheumatology patients treated with DMARDs in Saudi Arabia and found that respiratory tract and UTIs were the most prevalent [20]. The NOR‐DMARD study focused on RA and PsA patients taking TNFi treatment, reporting crude IRs of 4.17 (95% CI: 3.52–4.95) in RA and 2.16 (95% CI: 1.66–2.81) in PsA patients. The majority (37%) were respiratory tract infections [14]. A study by Ozen et al. showed an SI IR of 26.9 for TNFis, 23.3 non‐TNFi bDMARDs, per 1000 patient‐years. The SI risk with non‐TNFi bDMARDs vs. TNFi was not different [28]. The incidence of indicator of opportunistic infections due to bDMARDs was 23 cases per 1000 patient years, in a study by Leon et al. [32]. A 2018 Danish and Swedish Study explored the infection risks in RA patients treated with non‐TNFi bDMARDs (abatacept, rituximab, and tocilizumab). In this study, age and gender‐adjusted IRs of SI per 100 person years for abatacept/rituximab/tocilizumab were 7.1/8.1/6.1 for Denmark and 6.0/6.4/4.7 for Sweden, respectively [13]. Infliximab was associated with an increased risk of SIs compared with nonbiologic systemic therapies in patients with psoriasis in the United Kingdom and the Republic of Ireland. The incidence rates were significantly higher in the infliximab cohort (47.8 per 1000 person‐years, compared with 14.2 per 1000 person‐years in the nonbiologic systemic cohort [30]. Riek et al., in 2023, compared SI Risk of tofacitinib (tsDMARD) with bDMARDs. 67 SIs were identified whose occurrence overlapped with the 2182 patients’ study participation [15]. Interestingly, not all the studies identified increased risk of infections among patients treated with biologics. Holmgren et al., in 2023, studied IR of SIs among Swedish IBD patients before and after starting anti‐TNF therapy and found that it did not increase with anti‐TNF therapy. Instead, SIs seemed to decrease more than 1 year after initiation of anti‐TNF treatment. The IRs of SIs were 2.19 per 100 person years the year before and 2.11 per 100 person years 1 year after the treatment started [28].

This study lays down the basic demographic and clinical profile of SIs in patients treated with b/tsDMARDs in Qatar. It is unique in terms of the large number of drugs and disease conditions included. The IR of SIs was higher than all the above‐mentioned studies. Respiratory tract and UTIs were the most common in most studies like ours. However, there were significant variation in the profile of SIs from one study to the other because of the difference in patient cohort, drugs included, disease conditions included, and definition of SIs. Post hoc analysis of age categories revealed that the “above 70” group had significantly higher SI rates compared to the “18–35” (*p* = 0.001), “36–52” (*p* = 0.0003), and “53–70” (*p* = 0.0001) groups, highlighting the heightened vulnerability of elderly patients. Most studies showed an increased risk of SI among elderly and patients with multiple comorbidities. For example, in the Quartuccio et al. study [17], elderly patients (> 65 years) had a four‐fold increased risk of SIs compared to those < 40 years. This study is the closest to our study in terms of methodology that it was a retrospective study with 9 bDMARDs from different disease conditions included. In this study, sepsis was more frequent than skin and soft tissue infections. Also, the risk of infection was associated with comorbidities as measured by CCI, chronic exposure to glucocorticoids, or concomitant exposure to csDMARDs. We excluded patients with chronic glucocorticoid exposure, but there was no association with CCI or csDMARDs exposure.

According to current data and recommendations, TNFis (adalimumab, certolizumab, etanercept, golimumab, and infliximab) increase the risk of tuberculosis and fungal infections [23, 24, 27, 28, 30, 33]. *Mycobacterium tuberculosis* can survive within macrophages. TNFis disrupt the immune response against mycobacteria, leading to their reactivation or dissemination, resulting in active tuberculosis [27]. However, in our study we did not find any significant risk probably due to effective pretreatment screening and monitoring. 83.4% of the patients were screened with QuantiFERON tuberculosis test before starting biologics. 95% of those who were positive received chemoprophylaxis with a standard WHO regimen before initiation of the biologic. In our study, etanercept was associated with significantly increased risk of *S. aureus* infections. As a B‐cell depleting therapy, ocrelizumab may increase the risk of viral infections [29]. This is also reflected in our study where MS patients taking ocrelizumab had a significantly high risk of COVID‐19 SIs compared to other drugs. Hence, we strongly recommend that patients receive their updated COVID‐19 and annual influenza vaccines before being started on ocrelizumab. A study from the German biologic registry RABBIT (Rheumatoid Arthritis Observation of Biologic Therapy), reporting infection risk vs. TNFi, showed a 15% higher infection risk for tocilizumab [34]. However, such an association was not seen in our study due to the small population size of tocilizumab. In tofacitinib users, herpes zoster occurred at a rate of approximately 4% per year [15, 31]. However, most patients get treated as outpatients and were not considered as SIs.

Considering the low rates of vaccination among the patients in our cohort, it is important to discuss the latest guidelines [35, 36]. The 2019 EULAR recommendations for vaccinations in adult patients with autoimmune inflammatory rheumatic diseases (AIIRD) [36] advise yearly influenza vaccination to be strongly considered for the majority of these patients due to their susceptibility to severe influenza. Similarly, pneumococcal vaccination is strongly recommended for the majority to protect against pneumococcal infections [36]. According to Centers for Disease Control and Prevention (CDC) adult vaccination guidelines [37], adults aged 19 years and older with immunocompromising conditions, who have not previously received PCV13 or PPSV23, should receive a dose of PCV13 first, followed by a dose of PPSV23 at least 8 weeks later. Those who have previously received one or more doses of PPSV23 should be given a PCV13 dose 1 year after the last PPSV23 dose was received. As per EULAR [36], tetanus toxoid vaccination should follow general population guidelines; however, patients treated with B cell depleting therapy may need passive immunization. Hepatitis A and B vaccinations are advised for AIIRD patients at risk, with potential need for booster or passive immunization in specific scenarios. Herpes zoster vaccination may be considered for high‐risk AIIRD patients to prevent shingles. As per CDC [37], recombinant herpes zoster vaccine is recommended for adults aged 50 years and older, for those on low‐dose immunosuppressive therapy (e.g., less than 20 mg/day of prednisone), anticipating immunosuppression, or recovered from an immunocompromising illness. As per EULAR [36], vaccination against yellow fever is generally discouraged due to risks associated with live vaccines in immunocompromised patients. Human papillomavirus (HPV) vaccination is recommended for AIIRD patients, especially those with SLE, aligning with general population guidelines. Immunocompetent household members of AIIRD patients should receive vaccinations according to national guidelines, excluding oral polio vaccines to prevent potential transmission. Lastly, live‐attenuated vaccines are advised against during the first 6 months of life for newborns of mothers treated with biologics during the latter half of pregnancy to avoid the risk of severe infections from live vaccines [36].

Even though the current recommendation is that TNFi should be discontinued, at least temporarily, upon the occurrence of SI and the therapy should not be restarted until infection has been treated and clinical response is achieved [12], it is worthy to note that 22% of the patients in our cohort had no interruption in their biologic therapy and still had a favorable outcome. Hence it might not be mandatory to stop/change/interrupt the b/tsDMARD therapy on occurrence of an SI.

## 5 Study Design Limitations and Potential Biases

This study was a **retrospective observational cohort** conducted at a single healthcare institution, which inherently carries several limitations that could influence the findings.

### 5.1 Selection Bias

The inclusion of patients was based on their treatment with specific b/tsDMARDs as documented in the institution's EPR system. This reliance on EPR data introduces the possibility of selection bias, as it may exclude patients who were treated outside the HMC network. As a result, the data may underrepresent patients who experienced SIs but were treated elsewhere, leading to an underestimation of infection incidence.

### 5.2 Documentation and Data Completeness

As with many retrospective studies, there is a risk that not all relevant clinical events were documented thoroughly in the EPR system. This is particularly concerning for less severe infections that may not have required hospitalization or specific interventions, leading to potential underreporting of these events. While the study focuses on SIs that required hospitalization or were associated with more severe outcomes, it is likely that some milder infections went undocumented, skewing the study's infection profile.

### 5.3 Patient Selection

The exclusion criteria, which removed patients on long‐term glucocorticoids or csDMARDs, might also introduce bias. While these exclusions were necessary to isolate the effect of b/tsDMARDs, they could create a cohort that does not fully represent the typical population receiving DMARD therapies, particularly in real‐world settings where polytherapy is common. This may limit the generalizability of the study's findings to broader patient populations who are more likely to be on combination therapies.

### 5.4 Reliance on EPRs

The reliance on EPRs introduces potential information bias, particularly regarding the completeness and accuracy of the data recorded. EPR systems can vary in terms of data input quality, and some patient information may be missing, especially in cases where follow‐up data or vaccination records are incomplete. The study addressed missing data through multiple imputation techniques, but the inherent limitations of EPR documentation could still affect the robustness of the findings.

### 5.5 Inability to Establish Causality

Due to the observational nature of the study, causality between b/tsDMARD use and the occurrence of SIs cannot be established. The study highlights associations between certain b/tsDMARDs and infections, but these relationships are likely influenced by multiple confounding factors not fully accounted for in the retrospective design. Although efforts were made to adjust for confounders such as age and comorbidities, unmeasured variables may still play a role in influencing outcomes.

### 5.6 Potential for Residual Confounding

Although efforts were made to account for key variables such as age, comorbidities, and DMARD type, there remains a possibility of residual confounding from factors that were not measured or included in the analysis, such as adherence to treatment protocols, lifestyle factors, or the severity of underlying diseases. This may affect the interpretation of the associations found between b/tsDMARDs and SIs.

## 6 Recommendations

1. As skin and soft tissue infections were predominant and a significant proportion of them were caused by *S. aureus*, specifically MRSA, screening and decolonization should be done before starting b/tsDMARDs (especially etanercept).
2. The low vaccination rates for pneumococcal and influenza vaccines among biologic candidates in Qatar should be addressed immediately due to the high incidence of respiratory tract infections.
3. Vaccination guidelines discussed above should be strictly followed before starting patients on b/tsDMARDS.
4. Screening for latent tuberculosis infection (LTBI) should be performed before starting b/tsDMARDs, and chemoprophylaxis should be offered to patients diagnosed with LTBI to reduce the risk of progression to active tuberculosis.
5. Routine antibacterial or antifungal prophylaxis is not recommended for TNFi patients; however, in certain situations, such as a history of recurrent infections or specific comorbid conditions, the use of prophylactic antibiotics may be considered, guided by the patient's history and the treating physician's discretion.
6. Some live virus vaccines, such as yellow fever, varicella‐zoster virus or measles–mumps–rubella, may be contraindicated in patients receiving TNFi.
7. Patients should be educated about the signs and symptoms of infections and instructed to report any such signs promptly. This education should also emphasize the importance of avoiding exposure to contagious diseases and practicing good hygiene.
8. Once treatment has started, regular monitoring is necessary to detect any adverse effects early, including signs of infection. This monitoring should include periodic laboratory tests as indicated for the specific medication.

## 7 Conclusion

This comprehensive study conducted in Qatar has crucially filled gaps in our understanding of infection risks associated with b/tsDMARDs. It highlights the persistent risk of SIs across various patient demographics and the specific vulnerability associated with certain b/tsDMARDs, particularly adalimumab and infliximab. The research underscores the necessity of tailored patient management strategies, including vigilant monitoring and the strategic use of prophylactic measures to mitigate infection risks.

### 7.1 Implications for Clinical Practice

The findings of this study are particularly relevant for the clinical practice landscape in Qatar, where the use of b/tsDMARDs is prevalent. Clinicians should be alert to the elevated infection risks associated with these therapies and consider individual risk factors such as age and specific DMARDs in their treatment plans. This study supports the need for enhanced screening protocols, including regular monitoring for early signs of infection and the judicious use of vaccinations, especially in light of the low immunization rates observed among patients. Furthermore, given the study's insights, there is a strong case for integrating patient education programs that focus on infection risk awareness and preventive strategies.

### 7.2 Future Directions

To build on the findings of this study, future research should focus on longitudinal studies to track infection outcomes over longer periods and in larger populations to refine the risk profiles associated with specific DMARDs. Additionally, there is a need for randomized controlled trials to evaluate the efficacy of different prophylactic strategies in reducing infection rates among DMARD users. Expanding the research to include genetic and biomarker analyses could also provide deeper insights into individual susceptibility to infections, thereby enhancing personalized medicine approaches in the management of autoimmune diseases with DMARDs. Lastly, because this study highlights significant variations in infection risks across different DMARDs, developing a centralized registry for tracking and analyzing patient outcomes could vastly improve the clinical management and safety of these potent but potentially risky medications.

## Author Contributions

**Sreethish Sasi:** conceptualization, data curation, formal analysis, funding acquisition, investigation, methodology, project administration, writing – original draft, writing – review and editing. **Hamad Abdel Hadi:** conceptualization, investigation, methodology, project administration, writing – review and editing. **Masautso Chaponda:** methodology, writing – original draft, writing – review and editing. **Reem El Ajez:** data curation, methodology, writing – review and editing. **Mohamed Ataelmanan:** data curation, investigation, methodology, writing – original draft. **Sief Khasawneh:** data curation, investigation, writing – original draft. **Hind Saqallah:** data curation, investigation, writing – original draft. **Maisa Ali:** methodology, writing – review and editing. **Nabeel Abdulla:** methodology, writing – review and editing. **Javed Iqbal:** methodology, resources, writing – review and editing. **Ali S. Omrani:** investigation, methodology, supervision, writing – review and editing. **Muna Al Maslamani:** methodology, project administration, supervision, writing – review and editing. **Abdullatif Al‐Khal:** project administration, supervision, validation, writing – review and editing.

## Conflicts of Interest

The authors declare no conflicts of interest.

## Data Availability

The datasets generated and analyzed during the current study are not publicly available due to confidentiality agreements and privacy regulations but are available from the corresponding author on reasonable request. Requests for access to data should be directed to Dr. Sreethish Sasi at ssasi7@hamad.qa or sreethishsasi@gmail.com. Access will be granted to researchers who meet the criteria for access to confidential data as per institutional and ethical guidelines.
